# Supplementary material for: The Mental Health Outcomes and Cost Estimates of Korean Medicine for Anxiety Disorder Patients
Source: Healthcare (Basel). 2024 Jul 5;12(13):1345. doi: 10.3390/healthcare12131345 (PMC11241194; doi:10.3390/healthcare12131345)
Supplement: Supplementary file 1 [file healthcare-12-01345-s001.zip › healthcare-3017555-supplementary.pdf]

healthcare

MDPI

# Supplementary Materials

**Table S1.** Detailed description of anxiety-related scales

| Scale | | Description | Number of items |
|---|---|---|---|
| **Anxiety** | | | |
| State-Trait Anxiety Inventory (STAI) | STAI-X 1(state) | Current level of anxiety | 20 |
| | STAI-X 2 (trait) | The level of personality-based anxiety experienced in daily life | 20 |
| Beck Anxiety Inventory (BAI) | | The severity of anxiety with a focus on somatic symptoms designed to distinguish anxiety symptoms from depressive symptoms | 21 |
| **Anger** | | | |
| State-Trait Anger Expression Inventory State (STAXI) | STAXI-S (state) | Current level of anger | 10 |
| | STAXI-T (trait) | The level of personality-based anxiety experienced in daily life | 10 |
| Anger Expression Inventory (AXI) | AXI-K-I (anger-in) | Suppressing or hold anger in rather than expressing it | 8 |
| | AXI-K-O (anger-out) | The outward expression of anger toward other people or things either verbally or physically | 8 |
| | AXI-K-C (anger-control) | Ability to control angry feelings | 8 |
| **Depression** | | | |
| Beck Depression Inventory-II (BDI II) | | The level of depression | 21 |
| **Optimism** | | | |
| The revised Life Orientation Test (LOT-R) | | Measuring positive expectations towards the future | 10 |
| **Satisfaction** | | | |
| The Satisfaction with Life Scale (SWLS) | | Measuring current life satisfaction | 7 |
| The Life Satisfaction Expectancy Scale (LSES) | | Measuring anticipated satisfaction with future life | 7 |
| The Life Satisfaction Motivation Scale (LSMS) | | Measuring motivation for life satisfaction | 7 |

**Table S2.** The difference of outcomes for post treatment of weeks 4 between males and female

| | Range | Male (n=25) | | Female (n=42) | |
|---|---|---|---|---|---|
| | | Improvement from baseline (%) | p-value | Improvement from baseline (%) | p-value |
| **Anxiety** | | | | | |
| STAI X-1# | 20-100 | 15.3 | <0.001*** | 14.2 | <0.001*** |
| STAI X-2# | 20-100 | 7.7 | 0.003*** | 10.8 | <0.001*** |
| BAI# | 21-63 | 22.0 | <0.001*** | 22.7 | <0.001*** |
| **Anger** | | | | | |
| STAXI-S# | 10-40 | 7.3 | 0.022*** | 1.3 | 0.698 |
| STAXI-T# | 10-40 | 3.2 | 0.332 | 2.5 | 0.437 |
| AXI-K-I# | 8-32 | 4.2 | 0.125 | 5.6 | 0.046*** |
| AXI-K-O# | 8-32 | 5.5 | 0.007 *** | 1.6 | 0.455 |
| AXI-K-C | 8-32 | 3.3 | 0.862 | 0.4 | 0.865 |
| **Depression** | | | | | |
| BDI II # | 0-63 | 9.5 | 0.002*** | 12.5 | <0.001*** |
| **Optimism** | | | | | |
| LOT-R | 6-30 | 4.5 | 0.067 | 2.8 | 0.114 |
| **Satisfaction** | | | | | |
| LSMS | 5-35 | 0.4 | 0.891 | 0.3 | 0.864 |
| SWLS | 5-35 | 0 | 1.000 | 1.4 | 0.401 |
| LSES | 5-35 | 7.9 | 0.019*** | 5.2 | 0.057 |
| **Quality of life** | | | | | |
| EQ-5D | 0-1 | 3.5 | 0.042*** | 11.4 | <0.001*** |
| EQ-VAS | 0-100 | 7.0 | 0.021*** | 14.7 | <0.001*** |

AXI-K-C, Anger Expression Inventory Anger-Control; AXI-K-I, Anger Expression Inventory Anger-In; AXI-K-O, Anger Expression Inventory Anger-Out; BAI, Beck Anxiety Inventory; BDI II, Beck Depression Inventory-II; EQ-VAS, EuroQoL Visual Analog Scale; EQ-5D, Euro Quality of Life 5 Dimensions utility score; LOT-R, The Revised Life Orientation Test; LSES, The Life Satisfaction Expectancy Scale; LSMS, The Life Satisfaction Motivation Scale; STAI, State-Trait Anxiety Inventory, axis1 (X-1, State), axis2 (X-2, Trait); STAXI-S, State-Trait Anger Expression Inventory State Anger; STAXI-T, State-Trait Anger Expression Inventory Trait Anger; SWLS, The Satisfaction with Life Scale. # A lower score indicates a higher degree of symptoms. *** $p<0.05$

**Table S3.** Characteristics and outcomes of treatments combination

| Combination treatments | N (%) | STAIX1 (SD) | STAIX2 (SD) | EQ5D (SD) | EQVAS (SD) |
|---|---|---|---|---|---|
| Herbal medicine + Acupuncture + Psychotherapy + Aromatherapy + Family therapy + Chuna therapy | 10 (14.9%) | 9.00 (14.02) | 7.00 (10.38) | 0.18 (0.26) | 13.60 (14.44) |
| Herbal medicine + Acupuncture + Psychotherapy + Aromatherapy + Family therapy | 19 (28.4%) | 12.00 (8.97) | 8.16 (7.90) | 0.07 (0.08) | 17.11 (15.03) |
| Herbal medicine + Acupuncture + Psychotherapy + Aromatherapy + Chuna therapy | 16 (23.9%) | 8.88 (6.55) | 6.25 (5.07) | 0.12 (0.13) | 13.00 (16.19) |
| Herbal medicine + Acupuncture + Psychotherapy + Aromatherapy | 22 (32.8%) | 10.41 (7.42) | 6.95 (3.75) | 0.08 (0.10) | 16.59 (12.95) |

| EQ-VAS, EuroQoL Visual Analog Scale; EQ-5D, Euro Quality of Life 5 Dimensions utility score; SD, standard deviation; STAI, State-Trait Anxiety Inventory, axis1 (X-1, State), axis2 (X-2, Trait); Each value represents the mean difference between week 4 and week 1. ANOVA analysis indicated no significant differences among the groups.**Item** | **4 weeks treatment (n=67)** | | | | | **1-4 weeks treatment (n=36)** | | | | | **5~12 weeks treatment (n=36)** | | | | |
|---|---|---|---|---|---|---|---|---|---|---|---|---|---|---|---|
| | Mean | Standard deviation | Total | Minimum | Maximum | Mean | Standard deviation | Total | Minimum | Maximum | Mean | Standard deviation | Total | Minimum | Maximum |
| Number of visit | 5.1 | 1.6 | 339 | 2 | 9 | 5.2 | 1.5 | 188 | 2.0 | 9.0 | 5.9 | 2.9 | 214.0 | 2.0 | 15.0 |
| Consultation fee | 50,395 | 14,254 | 3,376,430 | 26,530 | 89,200 | 52,655.3 | 12,681 | 1,895,590 | 26,530 | 80,240 | 52,849 | 24,039 | 1,902,590 | 21,160 | 120,870 |
| **Costs of a diagnostic test** | | | | | | | | | | | | | | | |
| Pattern identification | 13,415 | 3,593 | 898,800 | 3,15`0 | 22,050 | 14,460 | 3,440 | 520,590 | 5,880 | 22,050 | 15,563 | 5,329 | 560,280 | 6,300 | 26,460 |
| Past psychiatric history | 8,153 | 221 | 546,240 | 7,920 | 8,550 | 8,128 | 225 | 292,610 | 7,920 | 8,550 | - | - | - | - | - |
| Heart Rate Variability | 3,574 | 73 | 239,440 | 3,440 | 3,650 | 3,581 | 74 | 128,940 | 3,440 | 3,650 | - | - | - | - | - |

| | | | | | | | | | | | | | | | |
|---|---|---|---|---|---|---|---|---|---|---|---|---|---|---|---|
| Personality test | 13,941 | 2,485 | 934,030 | - | 14,950 | 14,336 | 329 | 516,120 | 14,030 | 14,950 | - | - | - | - | - |
| **Costs of treatments** | | | | | | | | | | | | | | | |
| Acupuncture | 87,335 | 29,267 | 5,852,750 | 17,520 | 169,740 | 92,567 | 29,112 | 3,332,420 | 35,040 | 169,740 | 106,551 | 53,099 | 3,835,860 | 35,220 | 262,800 |
| Korean medicine psychotherapy | 82,789 | 27,993 | 5,546,870 | 40,800 | 215,070 | 80,510 | 17,383 | 2,898,390 | 40,800 | 129,220 | 76,618 | 39,550 | 2,758,270 | 24,900 | 180,300 |
| Family therapy | 13,998 | 22,565 | 937,850 | - | 79,650 | 19,346 | 26,280 | 696,480 | 0 | 79,650 | 11,393 | 21,326 | 410,150 | 0 | 65,920 |
| Chuna therapy | 55,705 | 80,007 | 3,732,200 | - | 263,790 | 64,173 | 90,177 | 2,310,260 | 0 | 263,790 | 66,520 | 100,575 | 2,394,750 | 0 | 351,720 |
| Herbal medicine# | 1,300,000 | 430,204 | 87,100,000 | 300,000 | 2,700,000 | 1,381,944 | 396,079 | 49,750,000 | 300,000 | 2,700,000 | 133,333 | 326,671 | 4,800,000 | 0 | 1,300,000 |
| Aromatherapy# | 59,188 | 19,968 | 3,965,600 | 23,400 | 124,200 | 58,516 | 16,127 | 2,106,600 | 23,400. | 92,700 | 58,277 | 32,720 | 2,098,000 | 15,200 | 151,000 |
| NHIS-covered cost | 329,325 | | | | | 349,761 | | | | | 329,497 | | | | |
| patient's out-of-pocket cost | 1,359,188 | | | | | 1,440,461 | | | | | 191,611 | | | | |
| Total | 1,688,513 | | | | | 1,790,222 | | | | | 521,108 | | | | |

**Table S4.** The costs of Korean Medicine for patients over weeks 4 and 12 (KRW)

National Health Insurance Service; NHIS

#Herbal medicine and aromatherapy were not covered by NHIS.

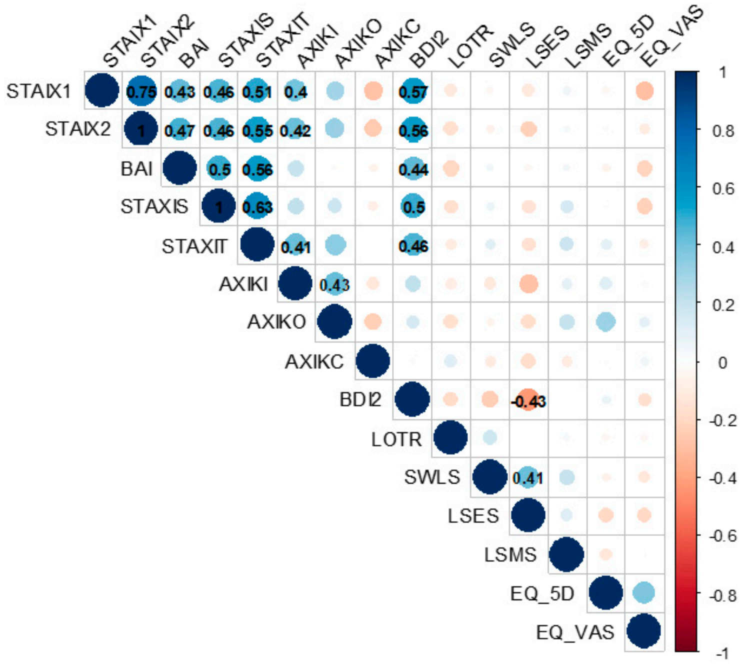


**Figure S1**. Correlation plot for the improvement in measurement scores over 4 weeks

Only correlation coefficients ≥0.4 are indicated in the plot.

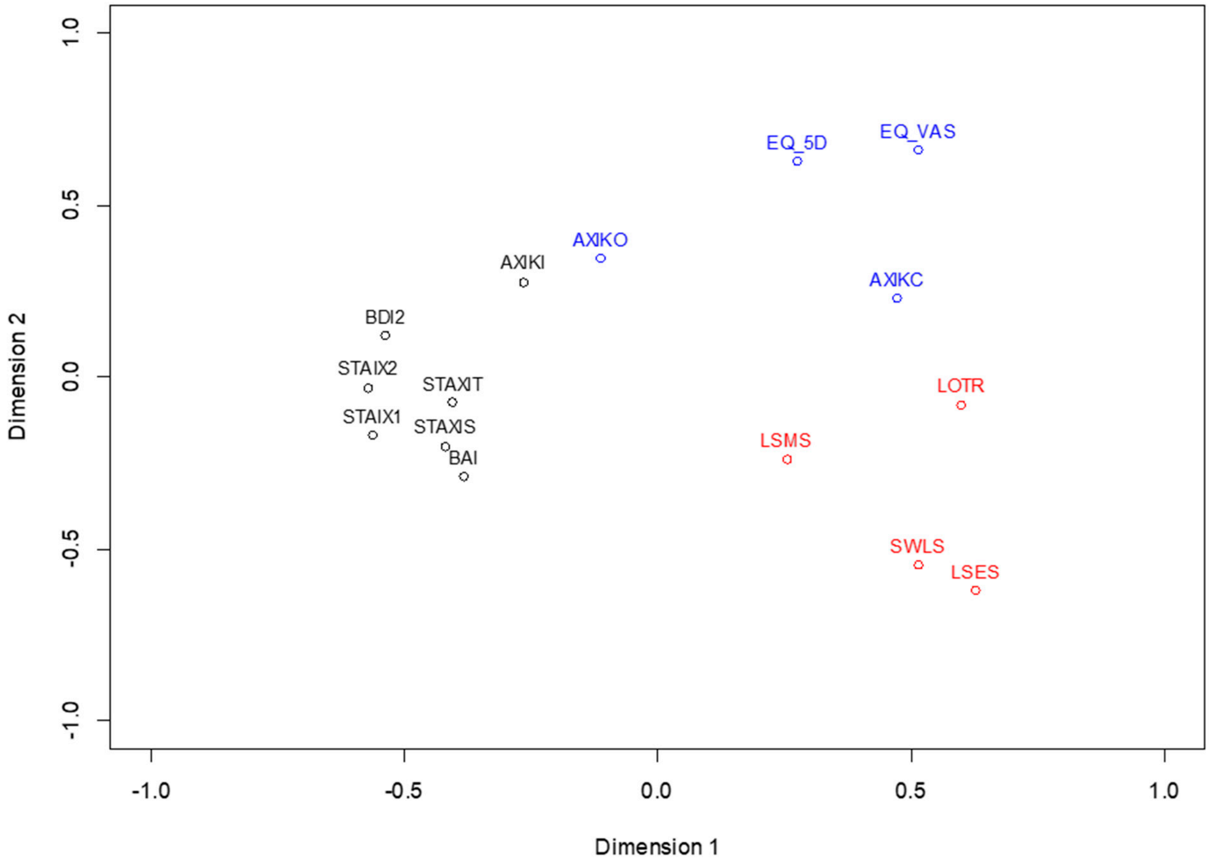


**Figure S2.** Plot of multidimensional scaling analysis, based on the clinic data
